# How many strata in an RCT? A flexible approach

**DOI:** 10.1038/bjc.2012.84

**Published:** 2012-03-13

**Authors:** P Silcocks

**Affiliations:** 1 CRUK Liverpool Cancer Trials Unit, University of Liverpool, Liverpool L69 3GL, UK

**Keywords:** controlled clinical trials, stratification, sample size

## Abstract

**Background:**

The need to allow for prognostic factors when designing and analysing cancer trials is well recognised, but the possibility of overstratification should be avoided by restricting the number of strata. The proposed method improves on existing guidance by being based on explicit principles and being more adaptable to circumstances, and should be of particular use to clinicians when designing a trial.

**Methods:**

Given a proposed sample size, a minimum allowable number in a stratum and an acceptable risk of observing fewer than this minimum, the number of strata can then be obtained by assuming a Poisson distribution for the number of observations per stratum. This can easily be programmed into Excel.

**Results:**

An example is given for a hypothetical typical trial of 250 patients, which for 80% power and 5% two-sided significance would correspond to a Cohen's effect size of 0.355 (about halfway between the ‘small’ and ‘moderate’ thresholds). To have a <1% risk of fewer than 10 patients in a stratum, no >13 strata should be considered. For a survival analysis with the same overall sample size but 170 deaths, no >9 strata would be prudent. In the context of a cancer trial this could easily be met by only two prognostic variables.

**Conclusion:**

The method proposed is flexible and based on explicit principles and may be applied in the design or analysis of both clinical trials and epidemiological studies.

The need to allow for prognostic factors when designing and analysing cancer trials has been recognised for many years (Simon, 1984), because the treatment effect that is being sought will often be smaller than the effects of prognostic factors. Stratified randomisation combined with a corresponding stratified analysis can counter this by increasing the precision of the estimated treatment effect and correcting for confounding bias due to uneven distribution of prognostic factors between treatment arms. In the field of cancer a wide range of such factors is known, reflecting characteristics of the host (age, performance status and comorbidity), the tumour (stage, grade and molecular markers) and the therapeutic environment (e.g., quality of surgery, or even surgeon workload as described by Stefoski Mikeljevic *et al* (2003)).

Typically in clinical trials this allowance is made by stratified randomisation (combined with permuted blocks to produce balanced treatment allocation within each stratum, which in turn will ensure that the distribution of observations across the strata will be the same for each treatment arm). The strata consist of the cross-classification of the categories (or levels) of a predefined subset of prognostic factors referred to as stratification variables.

Even if there is only one stratification variable some levels of it may be thin (i.e., have few observations). Even if this is not the case, because the overall number of strata is given by the product of the number of levels of each stratification variable, it is easy for the number of proposed strata to become disproportionately large relative to the sample size, especially in small trials. For example, assuming four levels for tumour stage and three for grade, these alone will generate 12 strata, whereas even if dichotomised, addition of performance status and age would result in 48 strata.

When the ratio of strata to sample size becomes too large, many strata may never be filled or hold only one or two patients. This is overstratification and can occur even if following advice that *‘*the use of more than two or three stratification factors is rarely necessary’ (European Medicines Agency, 1998), with choice of stratification variables being restricted to ‘…those known to be strongly associated with outcome’ (Green *et al*, 2003). Strata in which all patients receive the same treatment cannot contribute to a stratified analysis and are therefore wasted, with a consequent drop in power. If minimisation is proposed, constraints on the analysis still apply because the analysis should still account for the minimisation factors (Senn, 2004). The question is, therefore, not just which stratification variables a clinician should select from the bewildering assortment of prognostic variables that is available, but rather how many can be accommodated given the sample size. Naturally a stratified analysis assumes that the expected treatment effect is the same across strata. This can be tested for at the end of the trial, although power will be low unless such interaction was designed for, and may in any case be a function of the chosen analysis scale.

## Some existing advice

One rule states that the benefit of stratified randomisation requires the number of strata to be less than *N*/*B* where *N* is the total sample size and *B* is the block size (Hallstrom and Davis, 1988). Kernan *et al* (1999) go further and suggest that the number of strata be *N*/4*B* – the 4 being a safety factor – together with an average of 50–100 patients per stratum. These guidelines have two weaknesses: firstly the focus is on average number per stratum rather than on the ‘worst case’ and secondly there is an assumption of a block size, which would not apply to minimisation or urn randomisation.

Other guidance that also reflects the average number per stratum rather than the number of strata as such seems both more reasonable and less rigid: ‘while there are no set rules for the maximum number of strata permitted, practical considerations would suggest that there should be on average at least 10 patients per stratum, but at least 20 is better’ (Kelly and Halabi, 2010) but the use of both ‘on average’ and ‘at least’ is confusing.

## Materials and methods

Assume that the number per stratum is Poisson, with mean *X* given by *N*/*k* where *N*=sample size and *k*=number of strata and the minimum desired number per stratum is *m*.

This minimum number per stratum should occur rarely, say with frequency of ⩽1%. Using the square root transformation for a Poisson variate (with variance 0.25) we solve the following equation for *m*, where *z*_*α*_ is the standard normal deviate corresponding to the desired minimum frequency. 
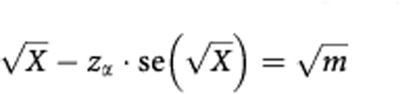


Substituting 0.5 for 
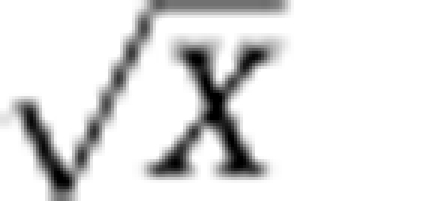
 and *N*/*k* for *X* and rearranging: 
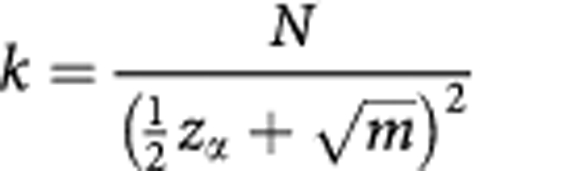


For a 1% risk of having fewer than *m* patients in a stratum, *z*_*α*_=2.33.

This is easily programmed into a spreadsheet.

What value is appropriate for *m*? For a two-arm trial a stratified analysis will ignore strata in which only one treatment is represented, so *m*=2 is an absolute lower limit. This is almost certainly too few unless its risk is made vanishingly small. Alternatively one could adopt the ‘10 or more observations/variable’ rule of thumb (van Belle, 2002) – the variable in question being the treatment indicator. For a two-arm trial this would be consistent with the recommendations of Kelly and Halabi (2010).

A point that is neglected in the existing advice cited here is that for trials employing survival outcomes, Poisson or binary responses, the effective sample size is the number of events and it is this number that should replace *N* in the formulae given here. This is a particularly important consideration in cancer trials (which mostly use survival analysis) because the prudent number of strata will be further reduced despite the large number of known prognostic factors.

## Results

Consider a hypothetically proposed two-arm study of 250 patients, with an average block size of 4. The *N*/4*B* rule would suggest 15 or 16 strata – but this would give an average of only 16 patients/stratum which is too low for Kernan *et al's* (1999) second criterion. To obtain an average of 56 patients per stratum a block size of 14 would be needed, but this is an excessive block size for a two-arm trial and would indicate only 4 strata at most. For a variable like tumour stage four strata could be entirely accounted for by only one prognostic variable and so is too restrictive.

By contrast suppose *m* is set at about 10 based on the ‘10 observations/variable’ rule. Note that while *m* may be a multiple of the smallest block size the proposed rule does not presuppose that allocation employs permuted blocks. With a 1% risk of a value smaller than *m*, a minimum of 10 per stratum would permit 13 strata. Given a block size of 4 and expecting all to be filled, *m*=12 would correspond to three average size blocks and indicate no more than about 11 strata.

Suppose, however, that the 250 patients was the overall sample size for a hazard ratio of 0.65 with a 5% two-sided significance at 80% power. The required number of events would be 170 (a maturity of follow-up of 68%) and 99% confidence for at least 10 events would permit only nine strata. Clearly the rule should not be too rigidly interpreted: if the rule gives a limit of 11 strata but 12 strata are defined, then 12 would be chosen.

## Conclusion

An advantage of the proposed method over those proposed by Hallstrom and Davis (1988) or Kernan *et al* (1999) is that it is not necessary to assume a particular block size. All that is required is the minimum tolerable number in a stratum and the risk of this occurring. This makes the rule applicable for those employing minimisation or urn randomisation methods as well as permuted block randomisation. A second advantage is that the focus is not the average number per stratum, but the distribution of numbers of patients across strata, with emphasis on the smallest value that is acceptable.

A Poisson distribution was assumed here for reasons of parsimony and prior ignorance. In reality of course it may be not be a good fit to the true distribution of frequencies in the strata. However, even if the marginal distribution is available from routine data sources or other publications, the joint distribution of prognostic factors is unlikely to be known. Even if a unit has previously carried out a trial on the same condition the required information may not be to hand because of variations in trial design because of advances in knowledge and variations in eligibility criteria. Nevertheless if even a rough estimate of the joint distribution of proposed strata does happen to be available, then simulation could be used instead of a Poisson assumption.

Once the formula proposed has been applied, a list of known prognostic factors may be drawn up in descending order of importance, with the corresponding numbers of levels. Often there is insufficient evidence as to the strength of the association with outcome, but in this respect the UICC publication ‘prognostic factors in cancer’ (Gospodarowicz *et al*, 2006) is a valuable tool for cancer researchers at least, that categorises prognostic factors as ‘essential’, ‘additional’ and ‘new/promising’. Once the cumulative product of the number of levels just reaches or slightly exceeds the recommended number of strata, the selection process can stop. For the purposes of defining the number of stratification variables it is probably better to ignore centre, unless there are only a few and the variation in outcome between them is comparable to that found with a prognostic factor such as tumour stage. Arguably such variation could be reduced by careful selection of centres. In addition, use should be made of standardised protocols and central review to improve inter-observer variation for variables that are known to have prognostic value, but which have high levels of misclassification, such as tumour grade (Bueno-de-Mesquita *et al*, 2010).

By rationing the number of proposed strata we can impose a more considered choice on both the number of stratification variables and the number of levels of each (Choi *et al*, 1995). No approach can be universally satisfactory but the proposed method is both adaptable and based on transparent principles. It should be a useful tool in planning RCTs.
